# Impact of *Rv0678* mutations on patients with drug-resistant TB treated with bedaquiline

**DOI:** 10.5588/ijtld.21.0670

**Published:** 2022-06-01

**Authors:** K. Kaniga, N. Lounis, S. Zhuo, N. Bakare, K. Andries

**Affiliations:** 1 Johnson & Johnson Global Public Health, Titusville, NJ, USA; 2 Janssen Pharmaceutica, Beerse, Belgium; 3 Janssen Research & Development, Titusville, NJ; 4 IQVIA, Durham, NC, USA

Dear Editor,

Bedaquiline (BDQ) is a diarylquinoline antimycobacterial that specifically inhibits mycobacterial ATP synthase. BDQ has been positively associated with treatment success and reduced mortality in multi-drug-resistant TB (MDR-TB),^1^–^4^ and is now approved in >60 countries. However, non-target-based mechanisms can result in decreased susceptibility to BDQ.^5^–^7^ Mutations in *Rv0678*, a transcriptional repressor of genes encoding the MmpS5-MmpL5 efflux pump, with concomitant upregulation of the efflux pump, MmpL5, account for cross-resistance between clofazimine (CFZ) and BDQ.^5^,^6^,^8^,^9^ The presence of *Rv0678* resistance-associated variants (RAVs) led to increased BDQ and CFZ minimal inhibitory concentrations (MICs) of 2- to 8-fold and 2- to 4-fold, respectively, in murine isolates, and increased BDQ MICs of 2- to 16-fold in clinical isolates.^6^,^7^ In murine isolates, a moderate BDQ MIC increase (3-fold) could be overcome by increasing the BDQ dose by 8-fold (from 6.25 to 50 mg/kg), but resistance of mutations yielding an 8-fold MIC increase could not be completely overcome.^6^ The efflux pump inhibitors verapamil (40 μg/mL) or reserpine (3 μg/mL) decreased the MICs of BDQ and CFZ in both drug-susceptible and drug-resistant isolates, but verapamil did not improve the bactericidal effect of BDQ in mice, and was unable to reverse efflux-based resistance in vivo.^6^ In many cases, *Rv0678* RAVs in clinical isolates are not associated with prior use of BDQ or CFZ, and do not result in MICs above the BDQ susceptible breakpoint (≤0.12 μg/mL).^7^,^10^ Mutations in clinically relevant *Rv0678* RAVs include single-nucleotide insertions, deletions and substitutions, large deletions, and random insertions of sequence elements.^6^ Previous studies have shown treatment failure on a BDQ-containing MDR-TB regimen, with emergence of *Rv0678* RAVs.^8^,^11^–^13^ However, there is little evidence from controlled trials on whether acquisition of *Rv0678* RAVs results in treatment failure, or if treatment failure results in acquisition of *Rv0678* RAVs, or if patients with *Rv0678* RAVs at baseline are more likely to fail BDQ treatment.

In an ad-hoc analysis of two Phase 2b BDQ clinical trial data, we investigated the impact on culture conversion rates of 1) presence of *Rv0678* RAVs in *Mycobacterium tuberculosis* isolates prior to treatment initiation; 2) *Rv0678* RAVs acquired during treatment; and 3) baseline BDQ MIC values of wild-type and *Rv0678* isolates. In the 120-week TMC207-C208 Stage 2 (NCT00449644) and TMC207-C209 (NCT00910871) studies, BDQ was given for 24 weeks (400 mg once-daily for 2 weeks, then 200 mg three times a week for 22 weeks) with a background regimen of anti-TB drugs given for 18–24 months in TMC207-C208^1^ and up to 30 months in TMC207-C209.^2^ TMC207-C208 Stage 2 was a randomized trial involving 160 MDR-TB patients, including pre-extensively drug-resistant-TB (pre-XDR-TB), comparing the efficacy and safety of BDQ vs. placebo.^1^ TMC207-C209 was an open-label, single-arm trial involving 233 newly diagnosed or previously treated patients with MDR-TB (including pre-XDR-TB or XDR-TB) confirming the safety and efficacy of BDQ.^2^ Patients recruited to both studies had a broad range of characteristics, including many with known risk factors for delayed sputum conversion (e.g., HIV, diabetes and/or cavitary disease).^1^,^2^ Patients’ *M. tuberculosis* isolates were target-sequenced for previously described *Rv0678* RAVs using the Sanger method.^7^ Corresponding BDQ MICs were determined by the 7H11 agar-dilution method.^7^,^14^ Microbiological outcomes (sputum culture conversion rates, no overruling for discontinuation) were assessed at Week 24 and endpoint (Week 120). Protocols were approved by an independent ethics committee/institutional review board, and all patients provided written informed consent.^1^,^2^

In the pooled TMC207-C208 and -C209 analysis, patients with baseline isolates without and with *Rv0678* RAVs (*n* = 226) had comparable conversion rates at the end of BDQ treatment (Week 24) (148/188, 78.7% and 28/38, 73.7%, respectively; Fisher’s exact test *P* = 0.52) (Figure). In analyses performed for the subset of patients (*n* = 165) with ≥1 positive sputum culture post-baseline, patients with wild-type isolates who acquired *Rv0678* RAVs post-baseline were more likely to fail treatment than those who did not acquire *Rv0678* RAVs (Figure). In the subset of patients with ≤2 active drugs in their treatment regimen, the culture conversion rate among patients who remained wild-type post-baseline was significantly higher than those whose post-baseline isolates acquired *Rv0678* RAVs (39/54, 72.2% vs. 2/9, 22.2%; *P* = 0.0065), and similarly, in the partially overlapping subset of patients with ≤3 active drugs (130/155, 83.9% vs. 4/10, 40.0%, respectively; *P* = 0.0034). Similar findings were seen at endpoint (Week 120: ≤2 active drugs: 37/50, 74.0% vs. 5/10, 50.0%; *P* = 0.1491; ≤3 active drugs: 86/109, 78.9% vs. 5/11, 45.5%; *P* = 0.0231).

**Figure:**
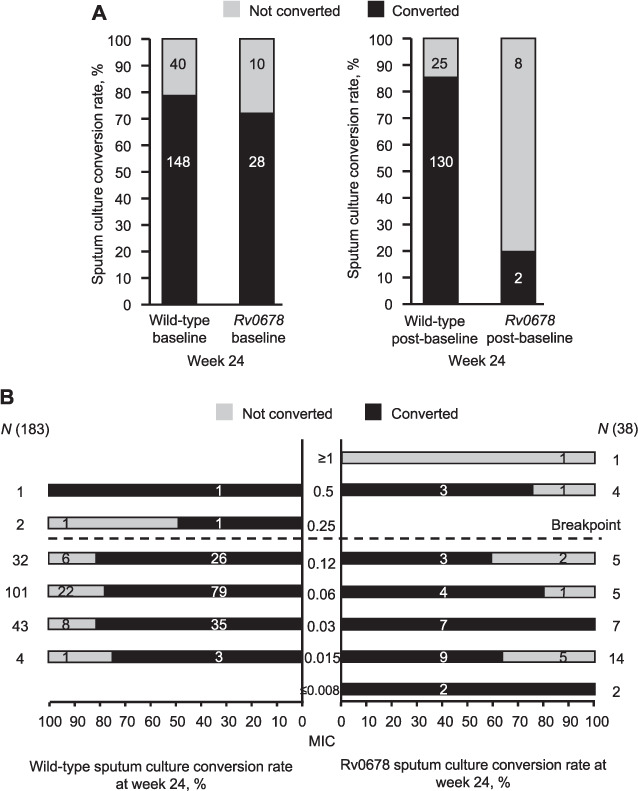
Effects of A) Rv0678 RAVs in baseline isolates and Rv0678 RAVs acquired during treatment, and B) baseline BDQ MIC for wild-type and Rv0678 baseline isolates on sputum culture conversion rates at Week 24 (no overruling for discontinuation) for BDQ-treated patients in the Phase 2b studies, TMC207-C208 Stage 2 and TMC207-C209. Sputum culture conversion was defined as two consecutive negative cultures from sputum samples collected at least 25 days apart. All intermediate cultures had to be negative as well. This condition was overruled when followed by a confirmed positive result, defined as two consecutive visits with positive sputum results, not taking into account intermittent missing/contaminated results. In the no-overrulingfor-discontinuation analysis, discontinuation information was not taken into account (patients who converted, then discontinued afterwards were considered as converted). In A), the numbers in the bars represent the actual number of isolates in each category (Week 24, P = 0.52 for culture conversion rate with baseline wild-type isolates vs. baseline Rv0678 RAVs; Week 24, P < 0.0001 for culture conversion rate with post-baseline wild type isolates vs. post-baseline Rv0678 RAVs). In B), the numbers in the bars represent the actual number of isolates in each category. RAV = resistance-associated variant; BDQ = bedaquiline; MIC = minimal inhibitory concentration.

For patients with BDQ MICs below the breakpoint, there was no correlation with culture conversion at Week 24 (Figure). For patients with BDQ MICs above the breakpoint, there were insufficient data to draw conclusions. Similar findings were seen at endpoint (Week 120). Based on this ad-hoc analysis in MDR-TB patients receiving BDQ in the Phase 2b TMC207-C208 Stage 2 and TMC207-C209 studies,^1^,^2^ the presence of *Rv0678* RAVs at baseline was not associated with poor treatment outcome.

We and others have described treatment failure on a BDQ-containing MDR-TB regimen coinciding with the emergence of *Rv0678* RAVs.^8^,^11^–^13^ However, given the apparent lack of effect of baseline *Rv0678* RAVs on treatment outcome, this is not a straightforward process to explain. We show that culture conversion can be achieved even in the presence of high BDQ MICs, provided the background regimen remains strong (it should be noted that the concurrent emergence of resistance to background drugs may also occur). This observation is made with caution because of the low incidence of baseline isolates with high MICs, which is to be expected for a drug with a new mechanism of action and limited clinical exposure at the time of conducting the Phase 2b studies. We did not evaluate any correlation between increased baseline BDQ MICs and treatment outcome in patients with wild-type vs. *Rv0678* RAVs at baseline. However, there are a variety of RAVs in *Rv0678* with variable effects on the BDQ MIC,^7^ and it is not possible to develop an algorithm to predict BDQ MICs based on specific *Rv0678* RAVs. Consequently, based upon the limited available information, sequencing for *Rv0678* RAVs is not useful to rule in BDQ susceptibility – it could only be used to exclude the likelihood of resistance due to *Rv0678* RAVs. This makes development of a rapid genotypic drug susceptibility test (DST) challenging. Thus, a standardized phenotypic DST method should be considered to determine susceptibility of *M. tuberculosis* to BDQ, especially among pretreated MDR-TB patients.^11^

In conclusion, no clear relationship was observed between the presence of isolates with *Rv0678* RAVs at baseline and poor treatment outcome. However, patients with wild-type isolates who acquired *Rv0678* RAVs post-baseline were more likely to fail treatment than those who did not acquire *Rv0678* RAVs.
